# Innovative Non-Thermal Technologies for Recovery and Valorization of Value-Added Products from Crustacean Processing By-Products—An Opportunity for a Circular Economy Approach

**DOI:** 10.3390/foods10092030

**Published:** 2021-08-29

**Authors:** Ana Cristina De Aguiar Saldanha Pinheiro, Francisco J. Martí-Quijal, Francisco J. Barba, Silvia Tappi, Pietro Rocculi

**Affiliations:** 1 Department of Agricultural and Food Science, Campus of Food Science, Alma Mater Studiorum, University of Bologna, Piazza Goidanich, 60, 47522 Cesena, FC, Italy; anacristin.deaguiar2@unibo.it (A.C.D.A.S.P.); silvia.tappi2@unibo.it (S.T.); pietro.rocculi3@unibo.it (P.R.); 2 Department of Preventive Medicine and Public Health, Food Science, Toxicology and Forensic Medicine, Faculty of Pharmacy, Universitat de València, Avda. Vicent Andrés Estellés, s/n, 46100 Burjassot, València, Spain; francisco.j.marti@uv.es; 3 Interdepartmental Centre for Agri-Food Industrial Research, Alma Mater Studiorum, University of Bologna, Via Quinto Bucci, 336, 47521 Cesena, FC, Italy

**Keywords:** chitosan, carotenoids, astaxanthin, non-thermal technologies, valuable compounds

## Abstract

The crustacean processing industry has experienced significant growth over recent decades resulting in the production of a great number of by-products. Crustacean by-products contain several valuable components such as proteins, lipids, and carotenoids, especially astaxanthin and chitin. When isolated, these valuable compounds are characterized by bioactivities such as anti-microbial, antioxidant, and anti-cancer ones, and that could be used as nutraceutical ingredients or additives in the food, pharmaceutical, and cosmetic industries. Different innovative non-thermal technologies have appeared as promising, safe, and efficient tools to recover these valuable compounds. This review aims at providing a summary of the main compounds that can be extracted from crustacean by-products, and of the results obtained by applying the main innovative non-thermal processes for recovering such high-value products. Moreover, from the perspective of the circular economy approach, specific case studies on some current applications of the recovered compounds in the seafood industry are presented. The extraction of valuable components from crustacean by-products, combined with the development of novel technological strategies aimed at their recovery and purification, will allow for important results related to the long-term sustainability of the seafood industry to be obtained. Furthermore, the reuse of extracted components in seafood products is an interesting strategy to increase the value of the seafood sector overall. However, to date, there are limited industrial applications for this promising approach.

## 1. Introduction

During the previous decade, the commercial production of processed fish and seafood products has significantly expanded with a consequent increase in by-product generation. Crustacean by-products represent a significant kind of by-product from seafood processing plants. Every year, approximately 6–8 million tons of waste is produced around the world following crustacean processing [1], mainly related to the recovery and conditioning of the edible parts of various crustaceans such as crabs, shrimps, and lobsters.

The major components of crustacean by-products (head and shells) are proteins (25–50%), followed by chitin (25–35%), minerals (15–35%), lipidic components (0.2–17%), and pigments [2,3]. Considering the increasing volumes generated and the length of the natural degradation process of shells, their efficient use is of paramount importance. The valorization of these residues, rather than their disposal or incineration, introduces the concept of circular economy to the seafood processing sector. As discussed by Ruiz-Salmón et al. [4] and Jacob et al. [5], among the challenges for increasing the sustainability of the European seafood sector, various approaches are being undertaken. The circular economy approach includes ensuring significant material savings throughout value chains and production processes but also generating extra value and unlocking economic opportunities.

Currently, crustacean by-products are mainly used for the recovery of chitin and chitosan, which is its deacetylated form. These compounds have been correlated to important biological activities, such as antioxidants, antimicrobial, and various other properties that could be exploited for food formulation to improve safety, quality, and shelf-life [6]. Moreover, other valuable components could be applied in the food and pharmaceutical industries, in particular, crustacean by-products can be exploited for the extraction of enzymes, products of protein hydrolysis (hydrolysates), lipids rich in polyunsaturated fatty acids (PUFA), and carotenoids could be also recovered from crustacean by-products [7,8].

The most common strategy to recover chitin and chitosan from crustacean by-products is still the use of chemical treatments (mainly involving strong alkali and acid), resulting, however, in negative economic and environmental consequences due to high costs and the production of harmful effluent wastewater [9]. Lately, the sustainable development of the environment and economy has gained increasing political and social interest, privileging the development of “green technologies” and the use of “green products” over conventional industrial ones. Moreover, concepts such as circular economy have been regarded as leading principles for eco-innovation, that aims a “zero waste” society and economy, in which waste and by-products are exploited as raw material for the development of new products and applications [10]. 

Innovative food processing technologies, based on non-thermal methods (i.e., ultrasound, high-pressure processing, pulsed electric fields, cold plasma, supercritical fluid extraction) have been proposed for use within the food industry including the extraction of valuable components from wastes and by-products [11]. The extraction of valuable compounds from crustacean by-products, combined with the development of novel technological strategies aimed at their recovery and purification, will allow for important results related to the long-term sustainability of the seafood industry to be obtained.

This review aims at providing a literature summary of the major crustacean by-products, the main emerging non-thermal process for their recovery, and the current applications in the seafood industry. First, the main potential valuable components recovered from crustacean processing by-products are described. Then, a summary of the most relevant research for the optimization of innovative non-thermal extraction technologies is reported regarding biomolecules from crustacean by-products obtained from their industrial processing. Finally, examples of the potential use and applications of the extracted compounds for quality improvement and shelf-life extension of the seafood products are summarized.

## 2. Crustacean By-Products as a Source of Valuable Compounds

**Figure 1:**
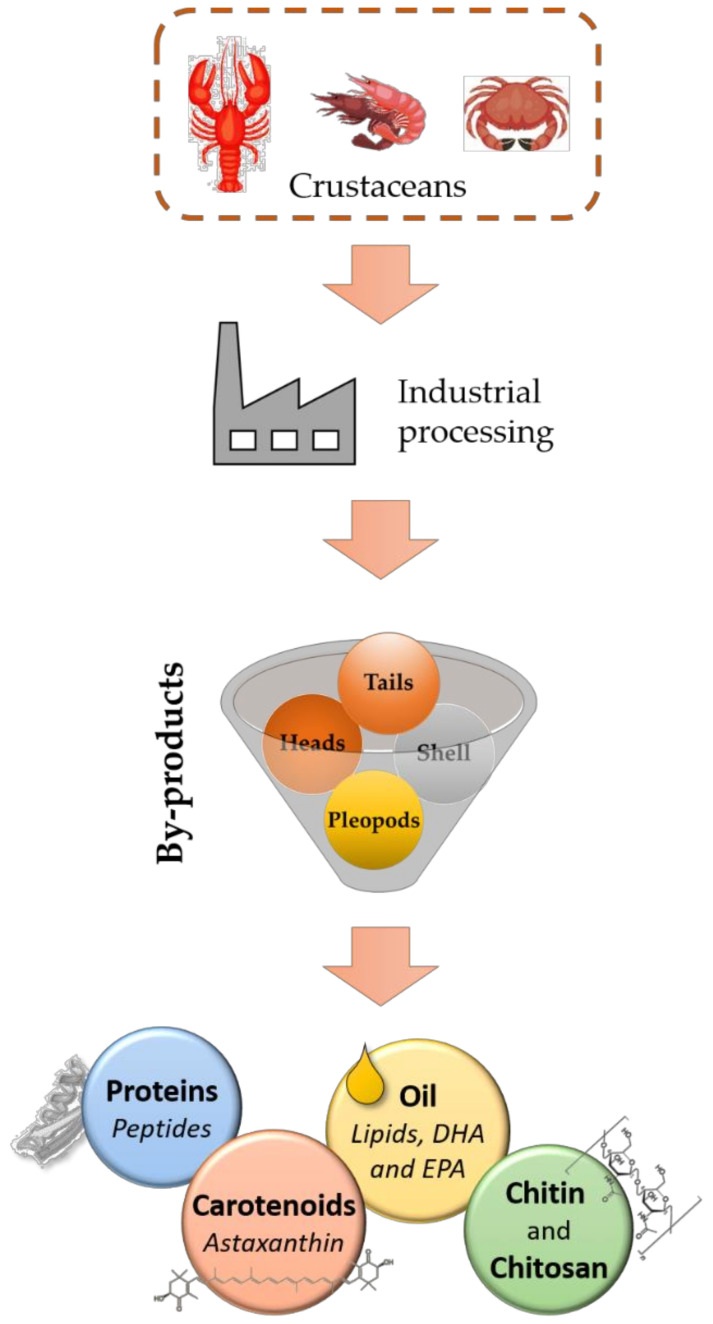
Valuable compounds derived from crustacean processing by-products.

Crustacean processing by-products (heads, shells, pleopods, and tails) contain several valuable compounds such as chitin, chitosan, carotenoids, lipids, and proteins, (Figure 1). These compounds show important biological activities, for instance, antioxidant, antimicrobial, and various other effects which can be exploited by the food industry with the aim of improving safety, quality, and shelf-life [12].

### 2.1. Proteins

The crustacean processing industry generates by-products rich in high-quality proteins and amino acids. Shrimp heads are characterized by high amounts of protein (50–65% dry weight) and are a very good source of essential amino acids, which is the reason they are used in aquatic animal feeds and are also included in livestock and poultry diets [13]. Lobster by-products are also extremely rich in protein and are characterized by an amino acid profile comparable to red meat, although higher in non-protein nitrogen (in the range from 10–40%) [14]. In lobster liver, proteins represent up to 41% of the dry matter [15], while the head retaining fleshy parts (body, breast, and leg) is approximately 20% of the total weight [16]. Additionally, the nutritional value of the lobster protein is greatly enhanced by its natural binding with a large amount of astaxanthin (295 μg/g), a powerful antioxidant to form a protein–carotenoid complex known as carotenoprotein [17]. Carotenoprotein isolated from shrimp by-products has shown high antioxidant activity, as well as being a rich source of essential amino acids and carotenoids [18,19], and has the potential for use as an additive to enrich foods and promote human health benefits [20]. With respect to other seafood species, proteins obtained from crustaceans are characterized by a higher content of some amino acids such as glycine, glutamic acid, arginine, and alanine, resulting in increased palatability compared to finfish proteins [14]. Moreover, on account of its optimal essential amino acid profile, the nutritional value of crustacean protein is similar or even higher compared to red meat [21] or soya bean [22]. For this reason, protein hydrolysates from shrimp by-products have been used for the fortification of different types of food products, such as biscuits [23] and bread [24]. Moreover, the functional properties of protein extracts from crustaceans have also been investigated for the production of an edible film [25]. 

The extraction efficiency of protein from crustacean by-products varies depending on the processing methods [26]. Hydrolysis is a common strategy for processing fish and shrimp waste with the aim of producing highly nutritive protein hydrolysates and recover bioactive molecules. Traditionally, protein hydrolysates from crustacean by-products have been obtained through the application of chemicals, microbial fermentation, and/or commercial enzymes [26]. However, chemical extraction leads to protein hydrolysates characterized by a higher degree of hydrolysis and lower efficiency of recovery, if compared to those obtained by enzymatic methods.

Enzymatic hydrolysis allows proteins to break down, altering their functional, chemical, and sensorial characteristics but maintaining the nutritional value [27].

Proteins can also be recovered from wash waters, e.g., from the washing process used to obtain surimi and from the peeling of shellfish and krill [28], but also from industrial cooking of crustaceans such as shrimp [29]. Apart from sarcoplasmic proteins and other water-soluble substances, a significant amount of functional myofibrillar protein can be found in waste waters. The recovery of these compounds is useful to reduce the amounts of contaminants and pollutants but also to valorize the by-product of industrial crustacean processing. There are many methods to recover these proteins, such as centrifugation, precipitation, micro- or ultra-filtration, and their combination. Ramyadevi et al. [30] optimized a process of aqueous two-phase system partitioning for the recovery and concentration of proteins obtained from the wash waters of shrimp. The functionality of the recovered proteins offers many possibilities for exploitation in further processes. For example, proteins recovered from shrimp surimi processing have been successfully exploited for the production of edible films [31].

### 2.2. Lipids

Crustaceans have appreciable proportions of ω-3 (omega-3) long-chain PUFA, particularly eicosapentaenoic and docosahexaenoic acid (EPA and DHA) [14]. The PUFAs are probably the most successful bioactive components isolated from marine sources because they have been widely recognized to be related to excellent health benefits [32].

Lipid content in crustacean by-products may be variable depending on the species, the fishery’s geographical location, and the kind of by-products. Recently, Albalat et al. [33] showed that oil recovered from the head waste of the Norway Lobster (*Nephrops norvegicus*) contains a higher proportion of EPA and DHA (15.0% and 8.3% of total neutral lipids, respectively, than krill oil (4.3% of EPA and 2.3% of DHA)). However, the content and profile of the recovered lipid are subjected to considerable variations according to the geographic location of the fishery and the seasonality.

Among crustacean waste products, cephalothorax and hepatopancreas have also been used as an excellent source of lipids with high PUFAs content [34,35] with a yield of approximately 2.7% and 11.6%, respectively. Although, in both waste types, PUFA represented the major lipid class and fatty acid profiles were different. The lipids from cephalothorax showed higher amounts of both DHA and EPA than those from hepatopancreas.

The lipid extract from crustacean cephalothorax processing by-products, containing high levels of PUFAs (including DHA and EPA), α-tocopherol, and astaxanthin, has recently been suggested to be added as a natural ingredient to food formulation where it could exert different effects, as a food coloring and as a functional ingredient [33,36]. Biological activities that have been attributed to lipids derived from shrimp by-products include antioxidant, anti-proliferative, anti-mutagenic, and anti-inflammatory effects [36,37,38]. 

Cholesterol may be a significant constituent of the lipid content of crustaceans. In the Pacific white shrimp (*L. vannamei*) by-products (cephalothorax, shells, tails, and pleopods), the lipid extract showed an appreciable amount of cholesterol (65 ± 1 mg/g) [39], in raw shrimp this value is usually greater than 100 mg/100 g of the edible portion of shrimp [40].

Lipids from crustacean by-products are oxidatively unstable, and the processes involved in their extraction may significantly affect their yield, quality, and stability [34]. The presence of astaxanthin and α-tocopherol seems to increase lipidic extract stability on account of their antioxidant properties [39]. However, their content was found to decrease during storage. Therefore, to expand their industrial application and utilization, recovery strategies that can improve yields without causing detriment to the quality of the extracted oil are necessary [41].

The most commonly used method for lipid extraction is based on the use of solvents; however, the high temperatures and the toxicity of solvents have increased the need for alternative extraction technologies. Alternative methods, to enhance efficiency in extraction such as the microwave, ultrasound-assisted extraction, supercritical fluid extraction, etc., represent a more environmentally friendly choice, requiring less use of less toxic chemical compounds (green technologies) [25]. In recent years, encapsulation of shrimp lipid extracts has also been investigated with the aim of increasing their stability and potential applications in food products. Various encapsulation techniques have been described for the oil obtained by crustacean by-products, such as complex coacervation [42], microencapsulation [43], spray-drying [44], and nano-liposomes [45,46]. Gómez-Guillén et al. [36] reported that the encapsulation process improved different functional properties, especially the antioxidant and anti-inflammatory properties and the water solubility, and maximized the bioaccessibility of astaxanthin. Based on the obtained results, the authors suggested the incorporation of the encapsulated extract with bioactive and technological functionalities, in different types of food products, for instance, meat or fishery products, soups, and sauces.

### 2.3. Carotenoids Pigments

**Figure 2:**
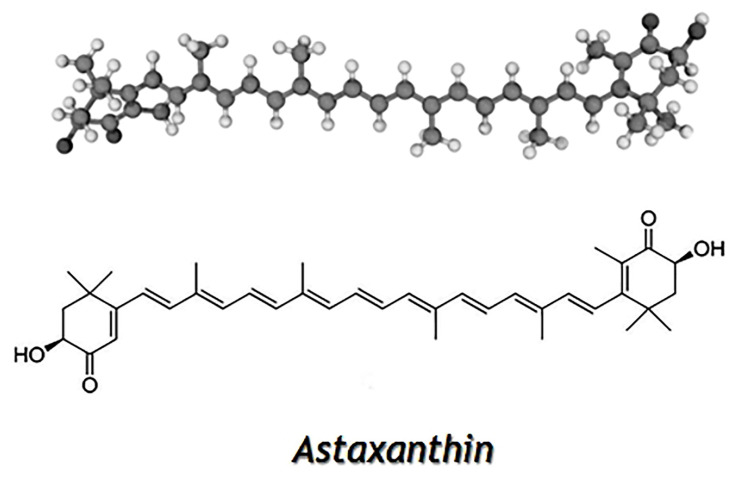
Chemical structure of astaxanthin.

Carotenoids are fat-soluble pigments found naturally in many marine products. Crustacean by-products represent important natural sources of carotenoid, among which astaxanthin (AX) is the major one. AX is composed of beta and beta-carotene-4,4′-dione with two hydroxy substituents in the positions 3 and 3′ (the 3S,3’S diastereomer) (Figure 2), and belongs to the xanthophyll family. In crustaceans, it is found in complexes with proteins and is the pigment that gives the typical animals’ color, and it is responsible for many biological properties such as protection from oxidative damage and the stimulation of growth and reproduction [47,48]. The content of AX in crustaceans can vary substantially, the variations observed in different shrimp species were in the range between 24 and 199 μg/g [49]. The observed differences could be due to variations in the amounts of carotenoid available in the feed, environmental conditions, and species, as well as due to the methods used for extraction. 

Generally, carotenoids are additives allowed in animal feed but also for food products and health supplements. The main application of AX is as a coloring agent added in the formulation of diets for various aquaculture species, in particular salmon, and it has been used for functional foods development [50], but it also finds various uses in the cosmetic and pharmaceutical industries [48]. The use of microencapsulation has shown great potential for the use of AX as a food ingredient for maintaining its coloring ability and overcoming some of its drawbacks such as odor [51] and improving its bio-accessibility and antioxidant capacity [52]. The antioxidant, activity of AX is ten times higher compared to other carotenoid pigments and approximately 100 times more than α-tocopherol [48].

The most used method for AX recovery is based on solvent extraction from wastes and by-products. A variety of solvents have been used, including hexane, acetone, isopropanol, ethyl acetate, methylethylketone, methanol, and ethanol, however, this method is considered costly, time-consuming, and not environmentally friendly [53]. Recently, other techniques aimed at increasing the process sustainably were investigated for carotenoid recovery, for example, microwave- and enzyme-assisted extraction methods, the use of supercritical fluid, and their combination. However, details regarding costs, efficiency, and environmental aspects related to these proposed strategies need to be carefully assessed [54].

### 2.4. Chitin, Chitosan, and Derived Compounds

Chitin, poly (β-(1→4)-N-acetyl-d-glucosamine) is a biopolymer, second in abundance only to cellulose. The major component of the exoskeleton of arthropods such as crustaceans and insects can also be found in some bacteria and fungi cell walls. The deacetylated form of chitin, mainly composed of glucosamine, 2-amino-2-deoxy-β-D-glucose, or (1→4)-2-amino-2-deoxy-D-glucose, is known as chitosan, which, contrasting with chitin, which is highly insoluble in most solvents, can be solubilized by decreasing the pH of the solutions. 

**Figure 3:**
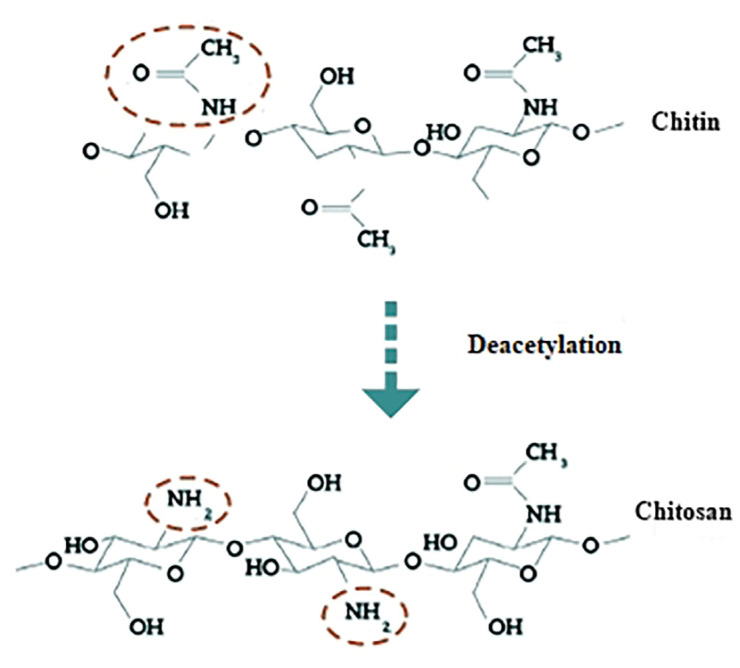
Deacetylation of chitin to chitosan.

Chitosan is characterized by the presence of three kinds of reactive functional groups as shown in Figure 3, an amino group in position C-2 and hydroxyl groups in positions C-3 and C-6. As well as the native forms of chitin and chitosan, it is possible to obtain modified forms, and all have a variety of applications [55]. To isolate chitin, first demineralization and deproteinization are applied, for both chemical and enzymatic treatments [56]. For residual pigment removal, it is possible to additionally apply a step of decolorization. Although different techniques have been suggested to purify chitin, a standard method is still lacking [57].

Crustacean exoskeletons are the main source of α-chitin aimed at commercial use on account of their high content and easy accessibility. These compounds, chitin, and its derivatives, gained increasing attention in various fields, from the pharmaceutical, biotechnology, biomedical to the food sector [6,57] on account of their various beneficial properties, as they are biocompatible, biodegradable, and safe. However, chitin has a limited application due to its insolubility in water and many solvents. Therefore, water-soluble derivatives are produced, chitosan being the most important. It shows interesting biological activities, such as antimicrobial and antioxidant characteristics that make it attractive for preservation as a possible alternative to chemical preservatives and for food packaging for producing edible antimicrobial films based on its good film-forming properties [58,59,60]. 

The deacetylation of chitin into chitosan can be obtained using chemical and enzymatic processes. For commercial purposes, the thermal-assisted chemical process that involves the use of a strong alkali (generally 40–50%, *w*/*w*, NaOH) coupled with high temperature is extensively used, due to the low cost and suitability for large-scale production. However, this process presents some drawbacks, such as a long reaction time, the use of high temperatures, low reproducibility of heterogeneous processes, which, in turn, leads to changes of chitosan characteristics, the possibility of depolymerization reactions caused by the use of highly concentrated alkali, and the production of high amounts of alkali wastewater that represent a potential environmental hazard [61]. The enzymatic method for converting chitin into chitosan is conducted using various chitin deacetylases (CDA) obtained by bacteria and fungi. However, previous studies show that deacetylation using CDA showed a lower degree of deacetylation (DD) than alkali treatment, indicating that these enzymes are not effective on insoluble chitins [62]. The enzymatic deacetylation reaction presents some limitations due to some chitin physical properties like crystallinity, solubility, and molecular weight [26]. Therefore, it is necessary to pretreat chitin before adding the enzyme, for increasing its accessibility to the substrate (acetyl group) and enhancing the yield of deacetylation [57]. 

To overcome the poor solubility of chitosan in water, hence widening its application, some processes can be exploited. Various polyphenol–chitosan conjugates have been developed, mainly for the development of films for food preservation. However, the study of their effects has been mainly carried out in in-vitro studies [63].

Through a chemical or enzymatic depolymerization process, chitooligosaccharides (COS) can be obtained from chitosan or chitin. They are characterized by shorter chain lengths and the presence of free amino groups in the unit of D-glucosamine; hence, they are soluble in water at a neutral pH, in contrast to chitin and chitosan, and present a low viscosity. These features make chitosan in its oligosaccharide form very attractive for use in the food and nutrition fields to enhance food quality and human well-being. COS industrial production is commonly obtained by acid hydrolysis aimed at cleaving the glycosidic linkages of chitosan. Nevertheless, this method leads to low yields and the production of a large quantity of monomeric D-glucosamine units [64]. On the other side, the use of the enzymatic process, based on non-specific enzymes, like proteases, lipases, and cellulases, and specific ones like chitosanases, is considered safe and easy to control [65]. However, the industrial application is limited by the high costs of enzymes, in particular the specific ones, such as chitinase. 

The antimicrobial activity of chitosan is highly variable and depends on many factors. Some are related to the chitosan molecule, e.g., the kind of chitosan, the molecular weight, and the deacetylation degree, while some extrinsic factors include the specific microorganism and the applied medium conditions, like pH, ionic strength, and types of solutes that can interact with chitosan hindering or blocking the reactivity of the active amine group. Considering the information obtained by the published literature, although antimicrobial properties of chitosan are variable, and many conflicting results have been presented, it seems widely accepted that the most sensitive group to chitosan are yeasts and molds, and then bacteria, Gram-positive and Gram-negative [66]. 

## 3. Innovative Non-Thermal Technologies for Recovery of Bioactive and Other Valuable Compounds from Crustacean By-Products

**Figure 4:**
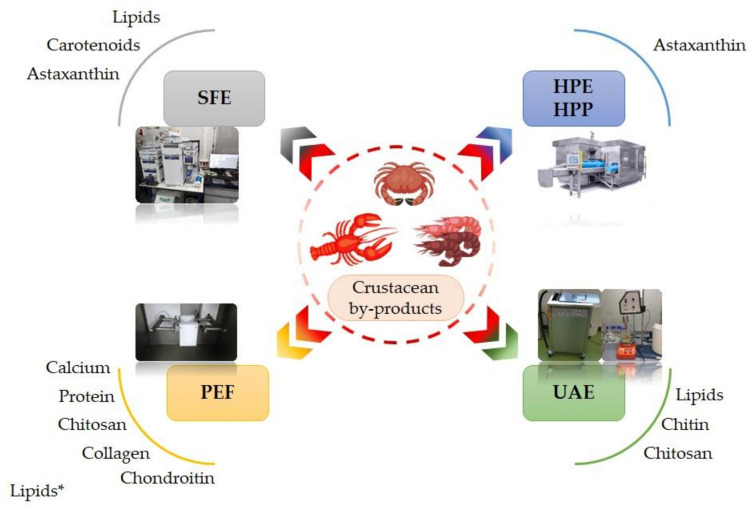
Main compounds extracted using non-thermal technologies. SFE: Supercritical Fluid Extraction; HPE: High-Pressure Extraction; HPP: High-Pressure Processing; PEF: Pulsed Electric Fields; UAE: Ultrasounds Assisted Extraction. * = used as a pre-treatment. The HPE/HPP picture was obtained from HIPERBARIC (Burgos, Spain) and used with permission.

Recently, the development of novel technological processes characterized by reduced energy consumption and impact on the environment increased the quality and safety of the final products, that can be applied for by-product valorization, have gained growing interest [67]. For these reasons, various modern non-thermal processes, such as supercritical fluid extraction (SFE), high-pressure processing (HPP), pulsed electric field (PEF), and ultrasound (US), have recently been suggested with the aim of shortening the processing time, increasing recovery yield, improving the product quality, and enhancing the functionality of extracts from crustacean by-products [68]. Figure 4 shows the main compounds extracted using non-thermal methods.

### 3.1. Supercritical Fluid Extraction (SFE) 

SFE technology is based on the separation of one component from a matrix, solid or liquid, using a supercritical fluid. Supercritical fluids are particularly suited for the extraction process because they are characterized by physicochemical properties that fall between those of a liquid and those of a gas, for instance, low viscosity, high diffusivity, and low surface tension [69]. An extensive variety of solvents can be used for SFE, such as carbon dioxide (CO_2_), nitrous oxide, ethane, propane, n-pentane, ammonia, fluoroform, sulfur hexafluoride, and water. However, CO_2_ represents the ideal solvent for application in the food industry, being inert, non-toxic, non-flammable, and cheap, therefore, it is the most used (conditions to obtain the critical state = 30.9 °C and 73.8 bar). Moreover, supercritical CO_2_ (SC-CO_2_) extraction is carried out at relatively low temperatures; hence, it is suited to heat-labile compounds, like carotenoids and lipids [70]. SC-CO_2_ shows good solvent characteristics for non-polar or slightly polar compounds and shows great affinity with oxygenated organic compounds of medium molecular weight [32]. Furthermore, CO_2_ creates a non-oxidizing atmosphere, hindering the oxidative degradation of compounds during extraction. Alternatively, it shows low affinity to polar compounds; hence, the use of co-solvents is suggested for their extraction as they increase their solubility in SC-CO_2_. 

SFE is a modern technology for extracting bio-compounds from various matrices that can be applied in the pharmaceutical and food industries. SFE has shown various advantages compared with traditional extraction processes, such as high yields, reduced processing times, and the use of solvents generally recognized as safe (GRAS), which make it a very popular green extraction method [71].

SFE has also been largely investigated for the valorization of food industry by-products [72]. For seafood by-products, much research is focused on the recovery of components producing high added value products, principally lipids and lipophilic components, such as carotenoids [73]. For the SFE of carotenoids, the five most critical parameters are processing temperature, pressure, time, CO_2_ density (solvent power) and flow rate, and entrainers concentration [54]. 

Table 1 reports examples of SC-CO_2_ for the recovery of lipids and astaxanthin from by-products derived from crustacean processing. Most of the published results showed that, when SC-CO_2_ was used alone, pressure and temperature did not impact the yield of oil extraction leading to the low quantity of recovered lipids and astaxanthin [70,74]. However, some authors have reported that adding co-solvents, generally ethanol or methanol, improved the extraction yields of both lipids and astaxanthin from by-products of crustacean processing. In this sense, Radzali et al. [75] investigated the use of different concentrations of different co-solvents (ethanol, water, methanol) for SFE of astaxanthin from the by-products of the shrimp *Penaeus monodon*. Lyophilized samples were extracted at a temperature of 60 °C and pressure of 20 MPa. The presence of ethanol maximized the yield (97.1% recovery compared to 100% with solvent extraction) of the total carotenoid (84.02 ± 0.8 μg/g) dry weight (DW). Sánchez-Camargo et al. [76] observed that at the conditions of 300 bar and 50 °C and using a 300 mL extractor with a constant solvent/feed (S/F) mass ratio (71.4), increasing the concentration of ethanol from 5 to 15%, allowed for the enhancement of the extraction of total lipid from freeze-dried shrimp by-products (*Farfantepenaeus paulensis*) by up to 136%. Considering the initial content in the waste material, lipids and astaxanthin were recovered up to 93.8% and 65.2%, respectively. Lipid recovery was significantly higher compared to other methods; 67% was recovered using only hexane as the solvent and 44.7% under the same conditions of temperature and pressure but without the use of a co-solvent.

Mezzomo et al. [77] evaluated carotenoid concentration through SFE from processing by-products of pink shrimp (*P. brasiliensis and P. paulensis*), taking into account the technical and the economic viability of the process. By-products were heat-treated, oven-dried, and milled, and 16 g were extracted in a 100 cm^3^ cell. Different parameters were investigated, such as the moisture content of the raw material (11.21% and 46.30%), temperature (40 °C and 60 °C), pressure (10–30 MPa), the solvent flow rate (8.3 g/min and 13.3 g/min), and nature of the co-solvent. The optimal conditions that allowed for astaxanthin yield maximization were the use of CO_2_ with the addition of 2% hexane: isopropanol solution (50:50) as a modifier, at 300 bar/60 °C. The cost analysis suggested the application of an SFE unit with 2 × 400 L vessels for 25 min extraction as the most lucrative process design. 

Amiguet et al. [78] evaluated the SFE efficiency on the recovery of PUFAs from the processing by-products of Northern shrimp (*Pandalus borealis*). They used a 100 mL extraction vessel for the processing of 10 g of an air-dried sample with a flow rate of 3–5 L/min. SC-CO_2_ extraction at 35 MPa and 40 °C resulted in deep red oil, with a high content of ω-3 PUFAs, in particular 7.8 ± 0.06% EPA and 8.0 ± 0.07% DHA.

Moreover, Nguyen et al. [15] optimized SC-CO_2_ lipid recovery with enriched PUFAs from Australian rock lobster (*Jasus edwardsii*) liver by using a 100 mL vessel for the treatment of 10 g of a freeze-dried sample at 35 MPa, 50 °C for 4 h (mass flow rate: 0.434 kg/h). Approximately 94% recovery was obtained and the extracted lipids were particularly rich in PUFAs (31.3% of total lipids), with a content four times higher compared to the one obtained by Soxhlet extraction (7.8%). In particular, DHA and EPA content was seven times higher.

Despite the various advantages of SFE, several concerns have been raised about the environmental and safety impact as well as the high energy consumption of the process. Other disadvantages of SFE include the limited sample size, extraction efficiency affected by matrix type, analyte type and moisture content of the matrix, and the high cost of SFE equipment [79]. Possible solutions investigated to increase its efficiency are the combination with other pre-treatments such as enzymatic treatment or the addition of co-solvents [73].

### 3.2. High-Pressure Extraction (HPE)

High-pressure processing (HPP) is a non-thermal food processing technique that involves the application of high pressure to solid or liquid foods with the aim of microbial inactivation but also of quality improvement [80]. Recently, the use of high pressure has been suggested for extraction purposes (High-Pressure Extraction—HPE) with the aim of reducing extraction time, solvent consumption, increasing extraction yields, and improving the quality of the obtained extracts [81].

HPE is based on the same principles of HPP (isostatic and Le Chatelier’s principles), the applied pressure levels usually range from 100 to 600 MPa, not affecting the covalent bonds, and the use of room or refrigerated temperatures prevents thermal degradation [82]. HPP produces physical damage to the plant tissue, cellular wall, membrane, and organelles, making cells more permeable to solvents, increasing the mass transfer rate, and facilitating the release of extracts. For this reason, HPE can be a useful strategy to valorize by-products facilitating the recovery of bioactive compounds. Indeed, compared to the conventional methods used such as thermal or solvent extraction, HPE is faster, allows for the increase of extraction yields, reduces impurities, and preserves the bioactivity of the extracted compounds, in particular, thermo-sensitive ones [81]. Another important advantage of HPE is its ability to use different solvents (and solvent ratios), with distinct polarities, enabling it to extract different components and to minimize the presence of impurities present [49]. 

HPE has been actively used to recover some biologically active substances from natural biomaterial; however, few researchers have evaluated the extraction from crustacean by-products (Table 2). Du et al. [82] studied the application of HPP for the extraction of astaxanthin from shrimp (*Litopenaeus vannamei*) by-products (shell and head) at ambient temperature, using ethanol as the extraction solvent, considering different variables such as the liquid-to-solid ratio (10 to 50 mL/g), applied pressure (0.1~600 MPa), and pressure holding time (0~20 min). The highest extraction yield (89.12 μg/g) was obtained by applying a pressure of 210 MPa for 10 min and a liquid-to-solid ratio of 32 mL/g. Similarly, Li et al. [49] studied the effects of pressure, holding time, different solvents (acetone, dichloromethane, and ethanol), and solvent-to-solid ratios for the HPE of astaxanthin from shrimp by-products at ambient temperature. The higher extraction yield (71.1 μg/g) was obtained in 5 min, using ethanol with a solvent/solid rate of 20 mL/g and a pressure range from 200 to 400 MPa. The antioxidant activity of the extracted astaxanthin was also found to be higher (EC_50_ = 81.54%) compared to that of conventional solvent extraction (EC_50_ = 45.31%). 

Recently, Irna et al. [83] studied the effect of HPE for astaxanthin extraction from six types of shrimp at 210 MPa, for 10 min with a solvent mixture of acetone and methanol (7:3, *v*/*v*), and compared it to conventional chemical extraction. Among the six species, the black tiger (*Penaeus monodon*) was the one with the higher amount with both extraction methods. Moreover, the same authors observed that the astaxanthin from shrimp carapace (*P. monodon*) extracted by HPE was characterized by higher antioxidant activity and a greater zone of inhibition against four bacterial strains (*E. coli*, *E. aerogenes*, *S. aureus,* and *B. subtilis*) compared to the chemically extracted one [84].

### 3.3. Pulsed Electric Fields (PEF)

PEF processing represents a novel, non-thermal method that has been shown as a potential tool to recover bioactive compounds from agri-food by-products [85]. Compared to conventional techniques, PEF offers several advantages such as non-thermal behavior, high selectivity, less time and energy consumption, and does not require any additional chemicals. PEF technology involves the application of a series of short high voltage pulses to a biological material (plant, animal, or microbial cells) placed between two electrodes. Pulses generally have a duration in the range from microsecond to millisecond, and a pulse amplitude that ranges from 100 to 300 V/cm to 20–80 kV/cm depending on the characteristics of the material. PEF treatment causes a phenomenon known as “electroporation”, related to the formation of pores in the cell membrane that facilitates the cell’s intracellular content release [86].

PEF treatment may be a promising method for the isolation and extraction of different components from seafood by-products such as calcium, chondroitin, collagen, chitosan, and protein [87,88,89]. However, the study of this technology for the extraction of compounds from crustacean by-products has been limited. 

Luo et al. [89] investigated the effect of the intensity of the electric field strengths up to 25 kV/cm (pulse duration (*τ*) of 20 μs, pulse frequency (*f*) of 1000 Hz, pulse number of 12, and flow rate of 100 mL/min) on the degradation of large molecular chitosan. From the traditional deacetylation of chitin, the obtained chitosan is characterized by high molecular weight (over 10^5^ Da) and low solubility in an aqueous solvent; hence, its application in food products results limited. The average molecular weight (MW) measured as the intrinsic viscosity value, of the PEF-treated chitosan, was reduced by increasing the intensity of the electric field. After the application of 15, 20, and 25 kV/cm, the MW decreased by 19.57%, 35.23%, and 45.19%, respectively, compared with the initial chitosan. At the same time, the authors observed significant damage to the crystalline region of the sample treated at 25 kV/cm, indicating a possible degradation of high MW. PEF treatments have shown a significant effect on the molecular structure of chitosan which may be responsible for the variation of its physicochemical and biochemical properties.

Gulzar and Benjakul [90] used a PEF pretreatment (to extract lipids from the cephalothorax of Pacific white shrimp (*Litopenaeus vannamei*) (electric field strengths in the range from 4–16 kV cm^−1^ and pulse number in the range from 120–240) in combination with an ultrasound-assisted process (UAE) that allowed for the maximization of lipid yield (30.34 g 100 g^−1^) and the reduction of lipid oxidation. Indeed, lipids from PEF-pretreated samples extracted using the UAE process showed an increased content of PUFAs and carotenoids, but peroxide value (PV) and thiobarbituric acid reactive substances (TBARS) were decreased. The authors suggested that the negative effects on lipid quality due to UAE might have been, to some degree, mitigated by PEF pretreatment; however, they did not put forward a possible mechanism for this observed phenomenon.

### 3.4. Ultrasound-Assisted Extraction (UAE)

The application of ultrasound (US) has proven to be a powerful method in food technology for processing, preservation, and extraction. US offers a significant advantage in productivity, yield, selectivity, reduced processing time, improved quality, the reduced presence of chemical and physical hazards, being considered environmentally friendly overall [91].

The major effects obtained by the application of US in a liquid medium are related to the cavitation phenomena and compression and decompression of molecules leading to the creation, enlargement, and implosion of microbubbles of gases dissolved in the liquid. The mechanical effects of US promote an increased penetration of solvent into the cellular material, an improved mass transfer thanks to micro-streaming, and the release of cell content due to the disruption of the biological cell walls [92].

The application of UAE in food processing technology improves the extraction of compounds from plant and animal tissues. The advantages of UAE include the reduction of extraction time and solvent consumption, improved reproducibility, simplified manipulation and work-up, and improved purity of the final product [91].

In recent years, several studies have demonstrated that UAE is a powerful method for extracting lipids from crustacean processing by-products due to its cavitation effect (Table 3). UAE increases the extraction yield of lipids and carotenoids; however, in some cases, it can lead to degradative processes such as lipid oxidation and hydrolysis that can be explained with the incorporation of oxygen and mechanical effects and with increased exposure of substrates to enzymes [93,94]. 

Several authors observed that UAE significantly improved the extraction yield of lipids and carotenoids from Pacific White shrimp (*Litopenaeus vannamei*) [90,93,94]. However, the UAE process caused, and increased, the lipid oxidation of lipids shown by higher peroxide values (PVs) and thiobarbituric acid reactive substances (TBARS), which was further increased using UAE with a continuous mode compared to the pulsed one. 

The addition of an antioxidant combined with UAE is a potential approach to reduce the disadvantages brought along by cavitation, in particular, the accelerated oxidation. As observed by Gulzar and Benjakul [90], pre-heating, along with 0.1% tannic acid addition, reduced lipid oxidation during the UAE of Pacific white shrimp.

The yield of lipids extracted from the lipid-containing solid residue (LSR) obtained from the protein hydrolysis of Pacific white shrimp cephalothorax was increased from 7.2 to 12% dry basis and carotenoid content increased from 5.7 to 8.6 mg/g of lipid when UAE was used at 80% amplitude for 10 min with a 30 s on-and-off pulse mode. 

Currently, UAE is widely used for the recovery of chitin and chitosan from crustacean by-products (Table 3). The ultrasound-assisted deacetylation (USAD) has been reported as an efficient process to produce chitosan. Birolli et al. [61], investigated the conversion of α-chitin from the cephalothoraxes of freshwater prawn (*Macrobachium Rosenbergii*) into chitosan applying the USAD. It was shown that the treatment of α-chitin suspended in 40% aqueous sodium hydroxide with high-intensity ultrasound irradiation strongly favored the N-deacetylation reaction, favoring the production of fully acid-soluble chitosan at high yield (>95%). Additionally, the USAD process allowed for the preparation of chitosan exhibiting a lower average degree of acetylation. 

Ngo and Ngo [95] evaluated the effects of low-frequency US on the heterogeneous deacetylation of chitin from the shell of white shrimp (*Penaeus vannamei*). At a low concentration of sodium hydroxide, below 45% (*w*/*w*), results showed that the US enhanced the deacetylation rate and, therefore, reduced the time of the reaction and improved the solubility of the chitosan. 

Kjartansson et al. [96] investigated the effect of sonication during chitin extraction from freshwater prawn (*Macrobrachium rosenbergii*) shells on the yield, purity, and crystallinity of chitin. Dry shells were suspended in 0.25 M HCl at 40 °C and sonicated for 0, 1, and 4 h at 41 W/cm^2^. Demineralized shells were suspended in 0.25 M NaOH at 40 °C and the samples were sonicated for 0, 1, and 4 h. It was found that the crystallinity indices and extraction yield of chitin decreased as the sonication time increased. The decrease in extraction yield was attributed to the leaching of depolymerized chitin during the washing step. The application of ultrasound enhanced the removal of proteins. Additionally, the degree of acetylation of chitin was unaffected by sonication, but the degree of acetylation of chitosan produced from sonicated chitin decreased from 70.0 to 61.4% after 4 h of sonicating the samples. 

Furthermore, some authors confirmed that the sonication of chitosan significantly reduces the MW of this polymer and has become an alternative method for degrading chitosan into low-molecular-weight chitosan (LMWC), chitosan oligomers, and glucosamine [97,98,99]. Intrinsic viscosity and average MW decreased exponentially with increasing sonication time, which is often desirable for its increase in antimicrobial activity and its use in pharmaceutical and biological applications.

### 3.5. Comparison of Technologies

Table 4 shows the main advantages and disadvantages of the considered innovative technologies used for the recovery of bioactive compounds in comparison with traditional ones. Generally, they all respond to the requirements of reduced processing times and reduced environmental impact. However, despite the numerous publications on the recovery of compounds from crustacean by-products, to the best of our knowledge, there are no publications comparing these new non-thermal technologies (SFE, HPE, PEF, and UAE) applied specifically for this aim. This is one reason it is not possible to select one of the investigated technologies as the optimal one, but others are because of the many processing parameters affecting the results and the numerous types of by-products derived from crustacean processing as described in the above paragraph. Indeed, as reported by Aoude et al. [100], a generic solution in terms of the recovery of high-value compounds from food waste does not exist; therefore, in each case, the optimal solution should be identified after individual study and optimization. 

Tsiaka et al. [102] compared the use of UAE with Microwave-Assisted Extraction (MAE) for the recovery of carotenoids from *Aristeus antennatus* shrimp. Both technologies obtained higher yields compared to traditional ones, but although using different solvents, results were similar for both. Moreover, the possibility to combine different techniques should also be considered.

## 4. Re-Use of Ingredients from Crustacean By-Products in Seafood and Food Products

The main compounds obtained from crustacean by-products are proteins and protein hydrolysates, oil-rich in PUFA, carotenoids, and in particular, astaxanthin and chitin derivatives, namely chitosan and COS. The recovery of flavors has also been investigated using the membrane filtration of seafood cooking effluents [103]. All these compounds find many applications in different sectors. The utilization of compounds obtained from shrimp processing by-products has been investigated in-depth, considering many applications in foods and feeds [104]. However, in the circular economy concept, their use in seafood-based products will increase the overall value of the seafood sector. In this section, some examples of the use of ingredients or compounds obtained from crustacean by-products for the formulation of seafood-based products are reported in Table 5.

Proteins recovered from shrimp can possess different functionalities that can be exploited in various food applications. The film-forming properties allows for the production of an edible coating [31] that can be applied to different kinds of products. Protein extracted and isolated from the muscles of *L. vannamei* were used for developing a coating that showed good potentiality to be applied for extending the shelf-life of fish-based products [105]. 

The protein recovered in the form of hydrolysates can be used as a flavoring and incorporated into fish-based foods or feed for aquaculture [106]. Moreover, the hydrolysates are also sources of biologically active peptides, with considerable potential in functional foods, nutraceuticals, and possibly supplements and/or as growth-stimulating agents in animal feeds [107]. Peptides derived from shrimp processing by-products, in particular, cephalothorax, shell, and tail have been demonstrated to exhibit antioxidative and cryoprotective effects in seafood [106,108,109] emphasizing their potential as alternative natural and safe preservatives with bi-functions, antioxidative, and cryoprotective effects, which can be used for maintaining the quality of seafood [108]. 

Lipid extract from shrimp waste could be used as a food ingredient due to its coloring capacity and antioxidant properties. Recent works have shown that the lipid extract obtained from shrimp (*Litopenaeus vannamei)* by-products is a promising food ingredient with multiple technological applications [25,42,52]. However, currently, there are a small number of studies that have focused on lipid extraction and practical application in food from this source. White shrimp (*Litopenaeus vannamei*) lipid extract rich in astaxanthin was encapsulated by ultrasonic atomization [110], achieving high encapsulation efficiency, antioxidant activity, and sensory acceptance when incorporated in the formulation of yogurt.

Chitosan and its derivatives are noted to have a wide range of functional properties that can be used for processing, preservation, and as a food additive to improve the safety, quality, and shelf-life of seafood. In recent years, chitosan has been researched extensively and shown to be effective in preserving the quality of various seafood products [58,60,111]. Several studies show that chitosan from crustacean by-products is characterized by an antimicrobial activity against a wide range of target micro-organisms on seafood products (Table 6). The effectivity of chitosan use as an antioxidant and antimicrobial on seafood products depends on the application method, type of seafood, concentration, and chitosan properties such as viscosity, particle size, molecular weight, and the degree of deacetylation [112,113].

Chitosan from crustacean by-products has also been used to improve the gelling properties of fish surimi products, the effect depending on the quality of the surimi, the type of chitosan, the concentration, and the gelling treatment [114,115]. Some studies suggest that the enhancing effect of chitosan on the gel formation of surimi could be due to the modification of the activity of the endogenous transglutaminase [116,117]. Chitosan films have been successfully applied as edible films and coatings for the packaging and protection of different seafood products [118]. The addition of protein concentrates rich in antioxidants like astaxanthin obtained from shrimp (*L. vannamei*) by-products has also been investigated [119,120]. The application of a film obtained by chitosan and added with a shrimp concentrate obtained by the cooking juice, achieved a novel product based on fish sausages and to extend its shelf-life up to 42 days [120]. Coating is the most popular application technique for chitosan followed by dipping, vacuum tumbling, spraying, and direct addition to the batter [58,111]. 

Chitooligosaccharide derivatives (COS) and chitosan nanoparticles possess potential applications in the seafood industry, due to their ability to protect food products against oxidative degradation, as well as preventing and/or treating free radical-related diseases [129,130]. Additionally, chitosan nanoparticles appear to be a promising agent for further improvement of chitosan coating efficiency. In a recent study, coatings containing chitosan nanoparticles were more effective in inhibiting microbial growth on silver carp (*Hypophthalmicthys molitrix*) fillets during refrigerated storage, than coating with normal chitosan [131]. Regarding the effect of chitosan nanoparticles from shrimp shells on the physicochemical properties of seafood-based products, it was reported that coating fish fingers with chitosan nanoparticles compared to commercial edible coating, reduced oil absorption by 11.86%, and increased moisture content by 18.09% during frozen storage at 18 °C [132].

## 5. Conclusions

The crustacean processing industry is a large source of by-products that can be a valuable source of nutraceuticals, bioactives, and functional compounds beneficial for human health. The recovery of by-products for beneficial health products offers economic and environmental benefits, thus, contributing to the concept of the circular economy in the seafood processing industry. This review has shown that innovative food processing technologies based on non-thermal concepts have the potential to be applied to the extraction of several biocompounds from crustacean by-products. These techniques are eco-friendly and safe and can increase the extraction yield reducing the processing time.

However, many of these techniques are poorly developed or tailored for crustacean by-product application and are lacking in standardization at the industrial scale. Moreover, crustacean by-products are diverse and complex. Considering these aspects, it is essential to define the appropriate extraction technology that allows for minimizing processing and maximizing quality for the target compounds.

The re-use of the extracted components in seafood products is a promising strategy to increase the value of the seafood sector overall. However, to date, there are limited industrial applications of this virtuous approach, particularly for chitin and chitosan valorization.

## Figures and Tables

**Table 1:** Supercritical fluid extraction (SFE) of crustacean by-products for the recovery of valuable compounds.

Species	By-Products	Compounds	Extraction Conditions	Optimum Condition	Yield and Characteristics of Products’	References
Australian Rock Lobsters (*Jasus edwardsii*)	Livers	Lipids	P: 25, 30, 35 MPa T: 50 °C CO_2_ flow rate: 0.434 kg/h Time: 240 min	35 MPa and 50 °C for 4 h:	94% of lipid yield, 4 and 7 times higher content of DHA and EPA, respectively, compared to those obtained by Soxhlet extraction	[15]
Tiger shrimp (*Penaeus monodon)*	Head and shells	Astaxanthin and other carotenoids	T: 60 °C, P: 20 MPa Co-solvents: ethanol, water, methanol, 50% (*v*/*v*) ethanol in water, 50% (*v*/*v*) methanol in water, 70% (*v*/*v*) ethanol in water, and 70% (*v*/*v*) methanol in water.	50% (*v*/*v*) ethanol in water	Carotenoid yield: 84.02 ± 0.8 μg/g dry weight (DW), Extracted astaxanthin complex: 58.03 ± 0.1 μg/g DW free astaxanthin content: 12.25 ± 0.9 μg/g DW	[75]
Pink shrimp (*Penaeus brasiliensis* and *Penaeus paulensis*)	Head, shell, and tail	Astaxanthin and other carotenoids	Moisture content (11.21–46.30%), solvent flow rate (8.3–13.3 g/min), T: 40–60 °C P: 100–300 bar co-solvent (hexane: isopropanol solution, 50:50, and sunflower oil)	Solvent: CO_2_ + 2% hexane: isopropanol solution, 50:50 Flow rate: 13.3 g CO_2_/min 11.21%: moisture content P: 300 bar T: 333.15 K	Global yield (amount of extract removed by the solvent and related to the solvent power, i.e., to the process temperature and pressure): 4.2 ± 0.2	[77]
Brazilian redspotted shrimp (*Farfantepenaeus paulensis*)	Head, shell and tail	Astaxanthin and ω3 fatty acid (EPA + DHA)	CO_2_/Ethanol Etahnol 5, 10 and 15% wt, P: 300 bar, T: 50 °C.	15% wt of ethanol.	93.8% and 65.2% for lipids and astaxanthin Total lipid extraction yield increased to 136% increasing ethanol from 5 to 15% wt.	[76]
Brazilian redspotted shrimp (*Farfantepenaeus paulensis*)	Head, shell and tail	Lipids, astaxanthin	P: 200–400 bar T: 40–60 °C	43 °C and 370 bar	Astaxanthin: 39% recovery Lipids yield similar under different conditions (1.74% to 2.21%) Possibility to fractionate oil	[70]
Northern shrimp (*Pandalus borealis*)	Head, shell and tail	Lipids (EPA+DHA)	Low P: 15 MPa, 50 °C Moderate P: 35 MPa; 40 °C	35 MPa; 40 °C	Total Fatty Acids: 795 mg/g Oil rich in ω-3 PUFAs (EPA:78 mg/g, DHA:79.7 mg/g)	[78]
Louisiana crawfish (*Procambarus clarkii*)	Shell and tail	Astaxanthin	T: 50–60–70 °C P: 13.8–22.4–31.0 MPa, Moisture content: freeze-dried 0–25–50%.	75 °C, 24.1 MPa, and 13% moisture.	Predicted maximum extractable astaxanthin: 207.6 mg/kg	[74]

P: Pressure; T: Temperature.

**Table 2:** High-pressure extraction (HPE) of crustacean by-products for the recovery of valuable compounds.

Species	By-Products	Compound	Extraction Conditions	Optimum Conditions	Yield and Characteristics of Products’	References
Pacific white shrimp (*Litopenaeus vannamei*)	Head and shell	Astaxanthin	P: 0.1–600 MPa, liquid-to-solid ratio (10 to 50 mL/g), and pressure holding time (0–20 min)	P: 210 MPa P holding time:9.2 min, liquid-solid-ratio: 32 mL/g	89.12 μg/g	[82]
Pacific white shrimp (*Litopenaeus vannamei*)	Shells	Astaxanthin	P: 0.1–600 MPa, holding times (0–20 min), different solvents (acetone, dichloromethane, and ethanol), and solvent to solid ratios (10–50 mL/g)	Ethanol, liquid to solid ratio of 20 mL/g and 200 MPa for 5min.	71.1 μg/g, better antioxidant activity in the extract than conventional solvent extraction	[49]
Rainbow Shrimp (*Parapenaeopsis sculptili*) Bird shrimp (*Metapenaeus lysianassa*) Giant river prawn (*Macrobrachium rosenbergii*) Shrimp (*Metapenaeopsis hardwickii*) Banana shrimp (*Penaeus merguiensis*) Giant tiger prawn (*Penaeus monodon*)	Head, shell, and tail	Astaxanthin	P: 210 MPa, time 10 min, solvent mixture of acetone and methanol (7:3, *v*/*v*).	Higher total carotenoid and astaxanthin yield obtained for *P. monodon*	Total carotenoid: 68.26 µg/mL astaxanthin yield: 59.9744 µg/gdw	[83]

P: Pressure.

**Table 3:** Ultrasound-assisted extraction (UAE) of crustacean by-products for the recovery of valuable compounds.

Species	By-Products	Compounds	Extraction Conditions	Optimum Conditions	Yield and Characteristics of Products	References
Pacific white shrimp (*Litopenaeus vannamei*)	Head	Lipids and carotenoids	Pulse and continuous Mode, sonication time (15, 20, 25, and 30 min), amplitudes of 50– 90%, 4 °C	80% amplitude with continuous mode, for 25 min.	50% yield, Extract richer in free fatty acids and higher oxidation level	[93]
Pacific white shrimp (*Litopenaeus vannamei*)	Head	Lipids and carotenoids	Frequency: 20 kHz, Power: 750 W, amplitudes: 60–100%	80% amplitude for 10 min at 4 °C	Lipid yield: 10–11 g/100 g, carotenoids yields: 8.6–8.8 mg/g lipid, higher lipid oxidation and hydrolysis	[94]
Giant river prawn (*Macrobrachium rosenbergii*)	Shell	Chitin	Demineralization in 0.25 M HCl (1:40 solid-to-solvent, *w*/*v*) at 40 °C, sonicated for 0, 1, and 4 h. Deproteinization in 0.25 M NaOH (1:15 solid-to-solvent, *w*/*v*) at 40 °C, sonicated for 0, 1, and 4 h	4 h	Lower content of proteins (7.45%) and deacetylation degree (61.4%)	[96]
Pacific white shrimp (*Litopenaeus vannamei*)	Shell	Chitosan	Deacetylation: NaOH (35%–65%, *w*/*w*), ratio of chitin (1:15, *w*/*v*), 80 °C, 360 min., frequency of 37 kHz and power of 300 W	Deacetylation rate improved with concentration of NaOH below 45% (*w*/*w*)	Higher solubility of chitosan	[95]

**Table 4:** Advantages and disadvantages of the considered technologies in comparison with traditional ones.

Technology	Advantages	Disadvantages	References
SFE	- High yields for carotenoids extraction - Reduced processing times - Use of solvents generally recognized as safe (GRAS), CO_2_ is inert, non-toxic, non-flammable, and cheap, high affinity for apolar compounds - Short extraction time and minimal usage of organic solvents	- Limited sample size - Extraction efficiency affected by matrix type, analyte type, and moisture content of the matrix - Difficult to optimize conditions - High cost of SFE equipment - Few commercial plants available	[71,101]
HPE	- Environmentally friendly - Rapid and highly efficient extraction - Reduced presence of impurities - Preservation of the bioactivity of the extracted compounds, in particular, thermo-sensitive ones - Ability to use different solvents (and solvent ratios) with distinct polarities	- High initial investment and capital costs - Batch or semi-continuous operation - No selectivity	[49,81]
PEF	- Non-thermal behavior - High selectivity - Less time and energy consumption - High yields for carotenoids extraction - Does not require any additional chemicals - Can be used in continuous mode	- Limitedly studied for the extraction of compounds from crustaceans by-products - Use can be limited due to the conductivity of matrix - High initial investment of PEF equipment - Limited extraction of lipophilic compounds	[85,87]
UAE	- Higher extraction yield or rate - Opportunity to use alternative (GRAS) solvents - Enhancing yield extraction of heat-sensitive components - Increase the yield of lipids	- Scale-up to industrial applications still needs to be explored and optimized - Can lead to degradative processes such as lipid oxidation and hydrolysis - No selectivity	[91,94]

**Table 5:** Application of bioactive compounds or products obtained from crustacean by-products in seafood products.

By-Product	Compond	Function	Seafood Product	Application	Findings	References
Shrimp Shell	Chitosan	Antioxidant	Rohu (*Labeo rohita*) fish sticks	Addition of 0.5%, 1%, 1.5%, and 2% of chitosan gel in batter for fish stick coating	Increase in chitosan gel concentration reduced oil absorption from 65–78%. Reduced total volatile basic nitrogen (TVBN), PV, and TBARS. Lipid oxidation decreased as the chitosan inclusion in batter increased.	[121]
Shrimp shell (*Metapenaeus dobsoni*)	Chitosan	Gelling	Surimi from Pangasius (*Pangasianodonhypophthalmus*)	Three different formulations by incorporating corn starch (10%) and chitosan (0.75%). A formulation containing only cornstarch (10%) was used as a control.	Reduction of total volatile basic nitrogen (TVBN), free fatty acids (FFA), peroxide value (PV), 2-thiobarbituric acid reactive substances (TBARS), and microbial count of the product during chilled storage. Extended the shelf-life of 17 days in comparison with the control of 10 days.	[122]
Shrimp cephalothorax, shell, and tail (*Penaeus monodon* and *Penaeus sindicus*)	Shrimp protein hydrolysate (SPH)	Antioxidant	Whole Croaker fish (*Johnius gangeticus*)	Dipping in various concentrations of SPH Solution (0.1%, 0.2%, and 0.5% (*w*/*v*) of 5 mg/mL concentration SPH solution)	Lowered TBA values of fillet and maintained yellowishness of skin color during 10 days of refrigerated storage at 4 °C and limited the increase of PV and FFA values.	[106]
Shrimp head (*Pandalus eous, Metapenaeus endeavouri, Penaeus monodon*)	Shrimp protein hydrolysate (SPH)	Cryoprotectant	Lizardfish (*Saurida spp*.) surimi.	Lizardfish surimi with 5% (dried matter) of any of the three SPH	Stabilized freeze-induced denaturation of myofibrillar protein and enhance gel-forming ability of surimi during frozen storage. Decreased the whiteness of all kamaboko.	[109]
Shrimp Shell (*Penaeus monodon), (Metapenaeus endeavouri)* *(Macrobrachium rosenbergii*)	Shrimp Chitin Hydrolysate (SCH)	Cryoprotectant	Lizardfish (*Saurida spp*.) surimi.	Lizardfish surimi with 5% (dried matter) of shrimp chitin hydrolysates	Delayed freeze-induced protein denaturation and increased the amount of unfrozen water in surimi stored at 25 °C for 6 months.	[123]
Crab shells	Chitosan	Antioxidant	Cooked comminuted flesh of herring (*Clupea harengus*)	Solutions with 50, 100, and 200 ppm of chitosan with a viscosity of 14, 57, and 360 cP, added directly on the minced fish	PV and TBARS were both reduced following treatment of the fish before cooking with 50, 100, and 200 ppm of chitosan 14, 57, and 360 cP. Inhibition of oxidation was concentrated-dependent and highest for chitosan 14 cP.	[112]
Prawn shell	Chitin and Chitosan	Gelling	Surimi from barred garfish (*Hemiramphus far*)	Chitin or chitosans with different degrees of deacetylation 65%, 83%, 88%, 99% DD) and concentrations were added to the surimi	Chitosan with 65.6% DD at 15 mg/g resulted in the maximum increases in both breaking force and deformation of suwari and kamaboko gels.	[114]

**Table 6:** Antimicrobial activity of chitosan from crustacean by-products in seafood products.

Species	By-Products	Microorganism Reduced and/or Inhibited	Application	References
Tuna fillets (*Euthynnus affinis*)	Shrimp shell	Aerobic plate count (APC) *Pseudomonas* spp. *Aeromonas hydrophila* *Salmonella enteritidis* *Klebsiella* sp. *Bacillus firmus* *Bacillus cereus* *Micrococcus* sp. *Escherichia coli* *Salmonella paratyphi* *Vibrio cholera* *Salmonella typhi* *Staphylococcus aureus*	Fillets were dipped in edible chitosan coatings (chitosan conc. 1%)	[124]
Smoked European eel (*Anguilla Anguilla*) stored under vacuum packaging (VP) at 4 °C	Crab shell	*Pseudomonas* spp. *Shewaella* spp. and yeasts/molds	Fillets were dipped in chitosan solution (2.0% *w*/*v*).	[125]
Pacific white shrimp (*Litopenaus vannamei*)	Shrimp processing by-products	Total bacterial counts (TBC) H_2_S-producer organisms Luminescent bacteria Total aerobic mesophiles *Pseudomonas* spp. *Enterobacteriaceae* *Lactic acid bacteria*	Chitosan coatings (chitosan conc. 2% *w*/*w*)	[126]
Fresh swordfish steaks (*Xiphia gladius*)	Crab shells	Total Viable Counts (TVC) *Pseudomonas* spp. H2S-producing bacteria Lactic acid bacteria Enterobacteriaceae	Chitosan with a concentration of 0.045% *w*/*w*, added by spraying it directly onto the product.	[127]
Salmon fillets (*Oncorhynchus nereka*)	Shrimp shells	Mesophiles, Psychrotrophs, coliforms, *Aeromonas* spp., and *Vibrio* spp.	Soaked in various concentrations of chitosan solutions (0.2%, 0.5%, or 1.0% in 0.1 N HCl, adjusted to pH 6.0 with 1 N NaOH)	[128]
